# Recent advances in natural and synthetic phosphonate therapeutics

**DOI:** 10.1016/j.mib.2025.102630

**Published:** 2025-07-12

**Authors:** Jerry Cui, Kou-San Ju

**Affiliations:** 1 Department of Microbiology, The Ohio State University, Columbus, OH 43210, United States; 2 Division of Medicinal Chemistry and Pharmacognosy, The Ohio State University, Columbus, OH 43210, United States

## Abstract

Phosphonate and phosphinate compounds — both natural and synthetic — have given rise to major families of therapeutics and agricultural agents. The antibiotic fosfomycin, the antivirals foscarnet and tenofovir, the bisphosphonates, and the herbicides phosphinothricin and glyphosate all belong to this compound class. The carbon–phosphorus bonds that define these molecules enable chemical mimicry of essential phosphate ester and carboxylate metabolites within metabolism, which is the foundation for their bioactivity. Here, we review examples of C-P compounds in drug discovery. In the first half, we highlight the ongoing development of two phosphonate natural products, both of which were initially discovered as antibiotics: fosmidomycin, which has undergone clinical trials as an antimalarial, and SF-2312, derivatives of which are currently being explored as chemotherapeutics. In the second half, we summarize how the C-P moiety has inspired chemical synthesis of new antimicrobials, immunomodulators, and targeted protein degradation agents.

## Introduction

Phosphonate and phosphinate (Pn) compounds, particularly natural products (NPs), are gifted with potent biological activity. The C-P moiety, which defines the compound class, facilitates chemical isosterism of carboxylates and phosphoesters and, therefore, chemical mimicry of critical intermediates distributed throughout metabolism [1]. This feature has endowed nearly all discovered Pn NPs with inhibitory biological activities. Notable examples include the clinically approved antibiotic fosfomycin, the widely used herbicide phosphinothricin (glufosinate), and the antimalarial candidate fosmidomycin. This reputation has led to a resurgence of interest in Pn NPs, enabling the development of genomics-driven methods, which have accelerated the discovery of novel chemical scaffolds. In the 65 years since the first Pn was isolated from biological material, more than 20 distinct groups of Pn NPs have been identified. Nearly a third of these have been discovered in just the past decade [2–6], and large-scale genome mining studies suggest that over a hundred groups of Pn await discovery from *Streptomyces* alone [2]. Here, we review current advances in the development of Pn therapeutics, both as NPs and synthetic variations of essential metabolites.

## Development of phosphonate natural product therapeutics

**Figure 1:**
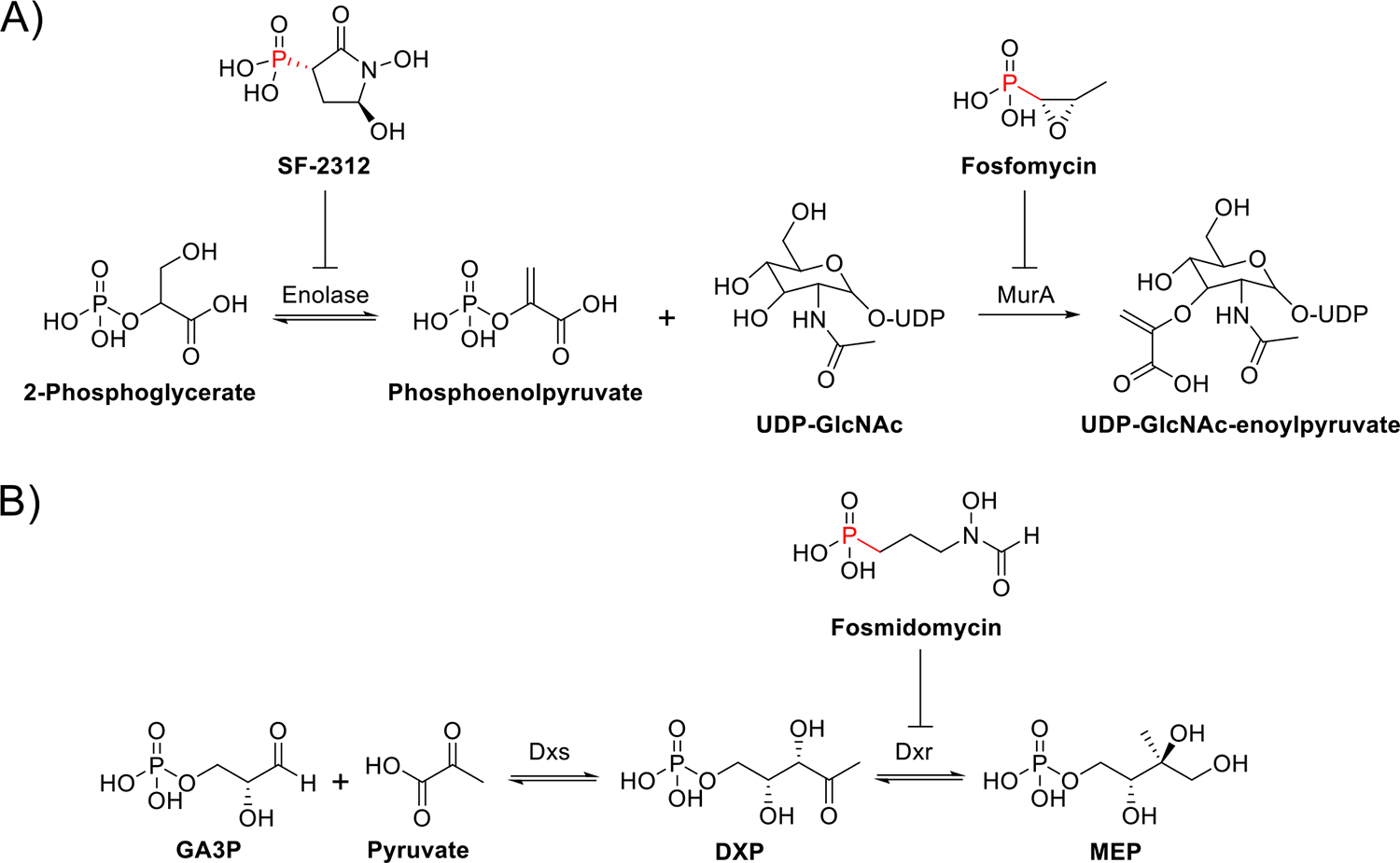
**(a)** SF-2312 inhibits enolase, preventing the conversion of 2-phosphoglycerate to phosphoenolpyruvate (PEP). Fosfomycin blocks cell wall synthesis by mimicking PEP and forming a covalent adduct with MurA, preventing the transfer of enoylpyruvate from PEP to UDP-*N*-acetylglucosamine (UDP-GlcNAc). **(b)** The first and rate-limiting step of the 2-C-methyl-d-erythritol 4-phosphate (MEP) pathway is the conversion of d-glyceraldehyde-3-phosphate (GA3P) and pyruvate to 1-deoxy-d-xylulose-5-phosphate (DXP) by DXP synthase (Dxs). Fosmidomycin blocks the second step of the MEP pathway, which is the interconversion of DXP and MEP by DXP reductoisomerase (Dxr).

Fosfomycin is the prototypical success story for Pn NPs as therapeutics. Discovered in 1969 through a screen for cell wall inhibition, fosfomycin was noted to be a broad-spectrum, bactericidal antibiotic [7]. It was subsequently found to act as PEP (phosphoenolpyruvate) analogue, irreversibly inhibiting MurA to block peptidoglycan biosynthesis (Figure 1a) [7]. Over the next few decades, screening programs continued to reveal bioactive Pn NPs. Examples include the herbicide phosphinothricin, the antifungal rhizocticin, and many antibiotics, such as fosmidomycin (Figure 1b) and SF-2312 (Figure 1a) [1]. Because of their bioactivity, Pn NPs have been commercialized at a rate orders of magnitude higher than NPs as a whole [8]. Here, we discuss two exciting areas of clinical development: the ongoing development of the antimicrobial fosmidomycin and the recent repurposing of the antibiotic SF-2312 as a novel chemotherapeutic.

### Fosmidomycin

**Figure 2:**
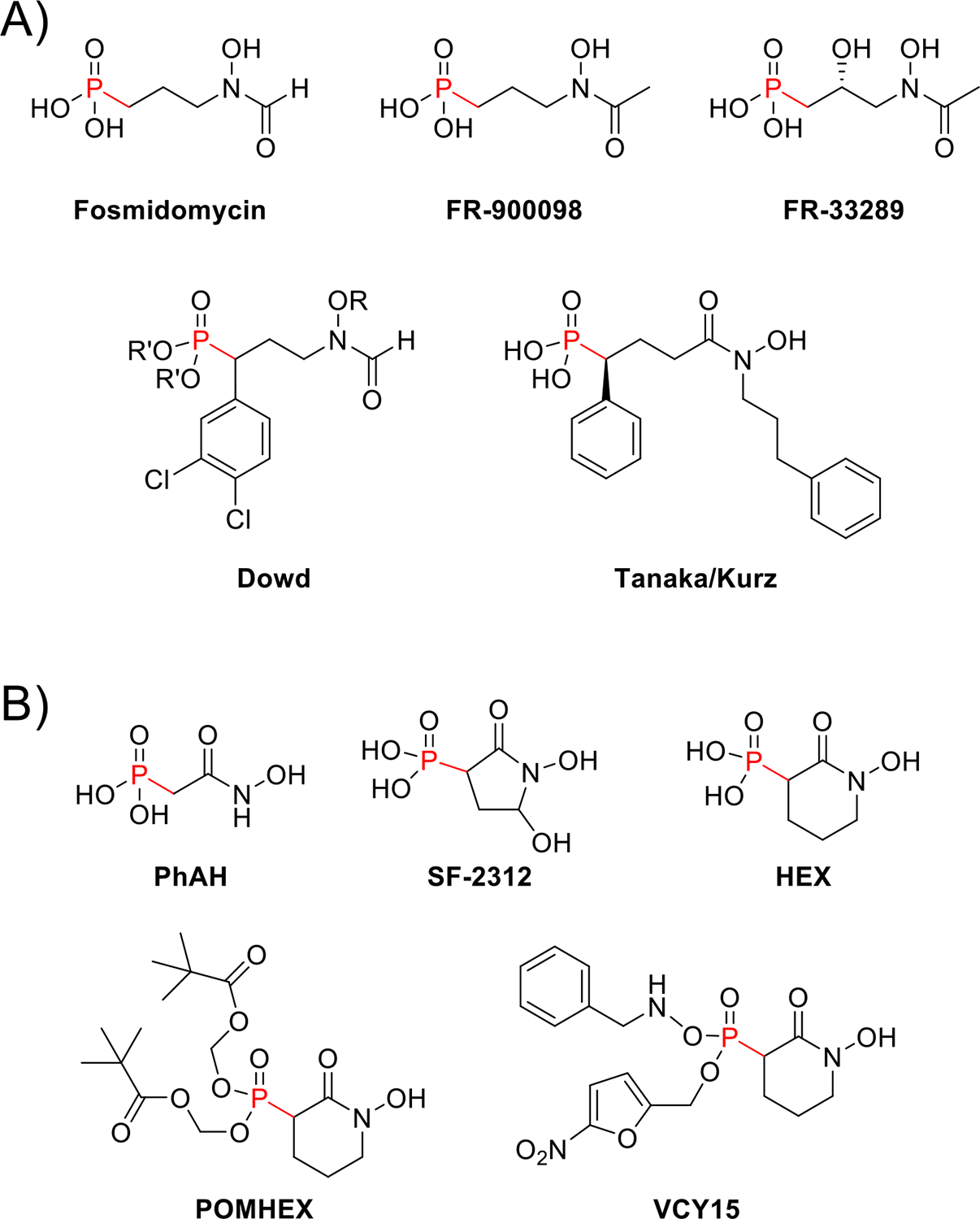
**(a)** Structures of fosmidomycin, FR-900098, and FR-33289, as well as the latest derivatives synthesized by the Dowd group and the Tanaka/Kurz groups. The Dowd group has experimented with various R groups (CH_2_-(2-napthyl), CH_2_-4-(1,1′-biphenyl), (4-iPr)Bn, and (CH_2_)_4_Ph) that allow extension into the NADH pocket of Dxr and different R′ groups (NH_4_ and POM) to create prodrugs. The Tanaka/Kurz groups focused on ‘reverse’ fosmidomycin analogues using a hydroxamate moiety rather than a retro-hydroxamate. Their latest compound features a *N*-phenylpropyl modification, which extends into a previously unknown subpocket of the Dxr catalytic domain. **(b)** Structures of phosphonoacetohydroxamate (PhAH), SF-2312, HEX, POMHEX, and VCY15. HEX is a ring-expanded derivative of SF-2312, while POMHEX is its bis-pivaloyloxymethyl (POM) prodrug. VCY15 is a recent nitroaromatic benzylamine prodrug of HEX, which is selectively bioreducible under hypoxic cellular conditions.

Antibiotic screening efforts in the 1970s and 80s resulted in the discovery of several related Pn NPs, including fosmidomycin (FR-31564), dehydrofosmidomycin (FR-32863), FR-900098, and FR-33289 (Figure 2a) [9]. Among these compounds, fosmidomycin displayed the lowest MICs (minimum inhibitory concentrations) and quickly advanced through preclinical development. Phase I studies confirmed that fosmidomycin was well-tolerated, and a phase IIa trial was conducted to assess its efficacy in Gram-negative urinary tract infections, but clinical development was halted for unclear reasons [10,11]. Decades later, the molecular target of fosmidomycin was revealed to be 1-deoxy-d-xylulose-5-phosphate reductoisomerase (Dxr), the second step in the nonmevalonate (MEP/DOXP) pathway for isoprenoids (Figure 1b) [9].

Recognizing the essential role of this pathway in *Plasmodium falciparum*, Jomaa et al. demonstrated that fosmidomycin and FR-900098 inhibited the *P. falciparum* Dxr, inhibited *P. falciparum* in culture, and cured mice infected with *P. vinckei* [12]. This quickly led to a small-scale human trial, where fosmidomycin therapy led to cure rates approaching 90% [13]. Unfortunately, subsequent clinical trials have failed to achieve this threshold, which the World Health Organization holds as the standard for acceptable efficacy [14]. Inadequate dosing may have been responsible for the failure of these trials [15]. Interestingly, FR-900098 has not entered clinical trials, despite exhibiting greater potency than fosmidomycin.

Deutsche Malaria GmbH is currently building upon previous combination therapies to develop a triple therapy of fosmidomycin, clindamycin, and artesunate (Fos-Clin-Art) for malaria treatment and prophylaxis [14]. The MEP pathway is essential to many other pathogens, including *Mycobacterium tuberculosis* [16], *Acinetobacter baumannii*, and *Klebsiella pneumoniae* [17]. While fosmidomycin has effectively inhibited the Dxr homologs of these organisms, *in vitro* inhibition has not resulted in *in vivo* activity due to difficulties with compound penetration, highlighting the need for pro-drug development.

Fosmidomycin has been the subject of more structure–activity relationship (SAR) exploration than any other Pn, and this topic has been well-reviewed by Knak et al.[14]. The Dowd group has contributed significantly and refers to fosmidomycin analogues as MEPicides [18]. Results of the SAR studies divide these compounds into three parts: the Pn moiety, a linker, and the retro-hydroxamate moiety. Most recently, the Dowd group has continued to develop prodrugs and bi-substrate inhibitors, which extend the retro-hydroxamate moiety to access the nicotinamide adenine dinucleotide phosphate (NADPH) cofactor binding pocket of Dxr (Figure 2a) [16,19]. Efforts by Tanaka and Kurz have similarly focused on prodrugs and *N*-substitutions, but with ‘reverse’ analogues of fosmidomycin, which have a hydroxamate rather than a retro-hydroxamate (Figure 2a). These analogues did not reach the NADPH binding pocket but instead bound to a previously unknown sub-pocket within the catalytic domain [20]. Binding within these two pockets has led to improved inhibition of Dxr and represents a promising avenue for lead optimization.

### SF-2312

Coincidentally, the other NP under clinical development also originated within the Japanese pharmaceutical industry. The cyclic Pn SF-2312 was isolated during a screen for antibiotics active under anaerobic conditions in 1986 (Figure 2b) [21]. There was little mention of the compound for the next three decades, until the Muller group began searching for γ-enolase (ENO2) inhibitors due to its essentiality in cancers with *ENO1* (encoding α-enolase) deletions [22]. While a potent inhibitor, phosphonoacetohydroxamate (PhAH, Figure 2b), had previously been synthesized, there was no structure of human ENO2 with PhAH. Thus, Leonard et al. began by docking and modeling PhAH with human ENO2. These experiments suggested that a cyclic derivative (deoxy-SF-2312) would increase rigidity and stabilize the bound configuration — and structure-based searches revealed a similar molecule in the chemical literature: SF-2312.

Synthetic SF-2312 inhibited ENO2, bound within the crystal structure of the enzyme, and exhibited selective toxicity against *ENO1*-deleted cells. Though unable to obtain enantiomerically pure compound due to spontaneous epimerization, only (*3S*,*5S*)-SF-2312 was bound within crystal structures. To determine whether this was truly the active enantiomer, methyl-SF-2312 was synthesized to examine stereochemistry at C3, generating *3S*- and *3R*-racemic mixtures. Again, only the *3S*,*5S* enantiomer of methyl-SF-2312 bound to ENO2 [23].

Following this discovery, Wright et. al re-examined the antibacterial properties of SF-2312, verifying that antibiotic activity was due to enolase inhibition [24]. Consistent with ENO2 studies, the crystal structures of *E. coli* enolase showed (*3S,5S*)-SF-2312 occupying the active site. As MurA acts downstream of enolase in peptidoglycan biosynthesis, the combination of SF-2312 and fosfomycin proved to be synergistic.

Over the past decade, the Muller group has proceeded to develop SF-2312. Guided by the crystal structure of ENO2, the cyclic ring was expanded to six atoms to generate HEX and a cell-permeable pivaloyloxymethyl (POM) prodrug termed POMHEX, which showed *in vitro* and *in vivo* (mouse xenograft) activity against *ENO1*-deleted cells (Figure 2b) [25]. However, POMHEX still demonstrated unfavorable pharmacokinetics, as rapid cleavage of a single POM exposes a charged species that cannot cross cell membranes. HEX requires two protecting groups for efficient cell permeation, which would ideally be designed to avoid premature cleavage and ensure selective release within tumors. The nitroaromatic benzylamine prodrug, VCY15 (Figure 2b), was cleverly designed to meet these criteria [26].

With VCY15, both protecting groups are cleaved by intracellular enzymes under select conditions. Intracellular nitroreductase activity is limited to hypoxic conditions, while intracellular phosphoramidases require a negative charge for hydrolytic activity. Therefore, VCY15 would remain intact until entering a hypoxic cell — such as a tumor cell. There, the nitrofuran is removed by nitroreductases, leaving an anionic benzylamine prodrug. This negative charge allows phosphoramidases to hydrolyze the benzylamine to release HEX. VCY15 demonstrated robust stability in human plasma and greater potency than POMHEX under hypoxic conditions. More recently, additional prodrug strategies with extensive SAR have been explored [27], and the pharmacokinetics and pharmacodynamics of POMHEX have been determined in mice [28]. The scope of these enolase inhibitors has also been expanded to *Trypanosoma brucei* and *Naegleria fowleri* [29,30].

The promising body of work, potent bioactivity, and ongoing clinical developments suggest that fosmidomycin-based therapeutics may soon become a reality. While the path may be longer for compounds derived from SF-2312, they offer potential cures for diseases lacking effective treatments, such as glioblastoma multiforme and primary amoebic meningoencephalitis. Together, these Pn NPs have essentially ‘unlocked’ new targets — the MEP pathway and glycolysis — for therapeutic development.

## Synthetic Pn bioisosteres

While the framework for Pn NPs as bioisosteres was established with the discovery of fosfomycin and its mechanism of action, the first reported use of Pn for human medicine predates these discoveries. In the 1960s, Herbert Fleisch and his collaborators discovered the critical role of pyrophosphate in calcium home-ostasis but were unable to use pyrophosphate or poly-phosphates to effectively inhibit abnormal calcification *in vivo* [31]. While searching for stable pyrophosphate analogues, the bisphosphonates were revealed to prevent bone resorption and inhibit pathologic calcification. This culminated in the use of the bisphosphonate etidronate to treat children with fibrodysplasia ossificans progressiva [32]. Further developments and discoveries have led the bisphosphonates to become the primary class of drugs for bone conditions [31]. Outside of their primary effect on bone metabolism, the bisphosphonates have also been utilized as bone-targeting agents, allowing for improved treatment of osteomyelitis [33].

### Antivirals

**Figure 3:**
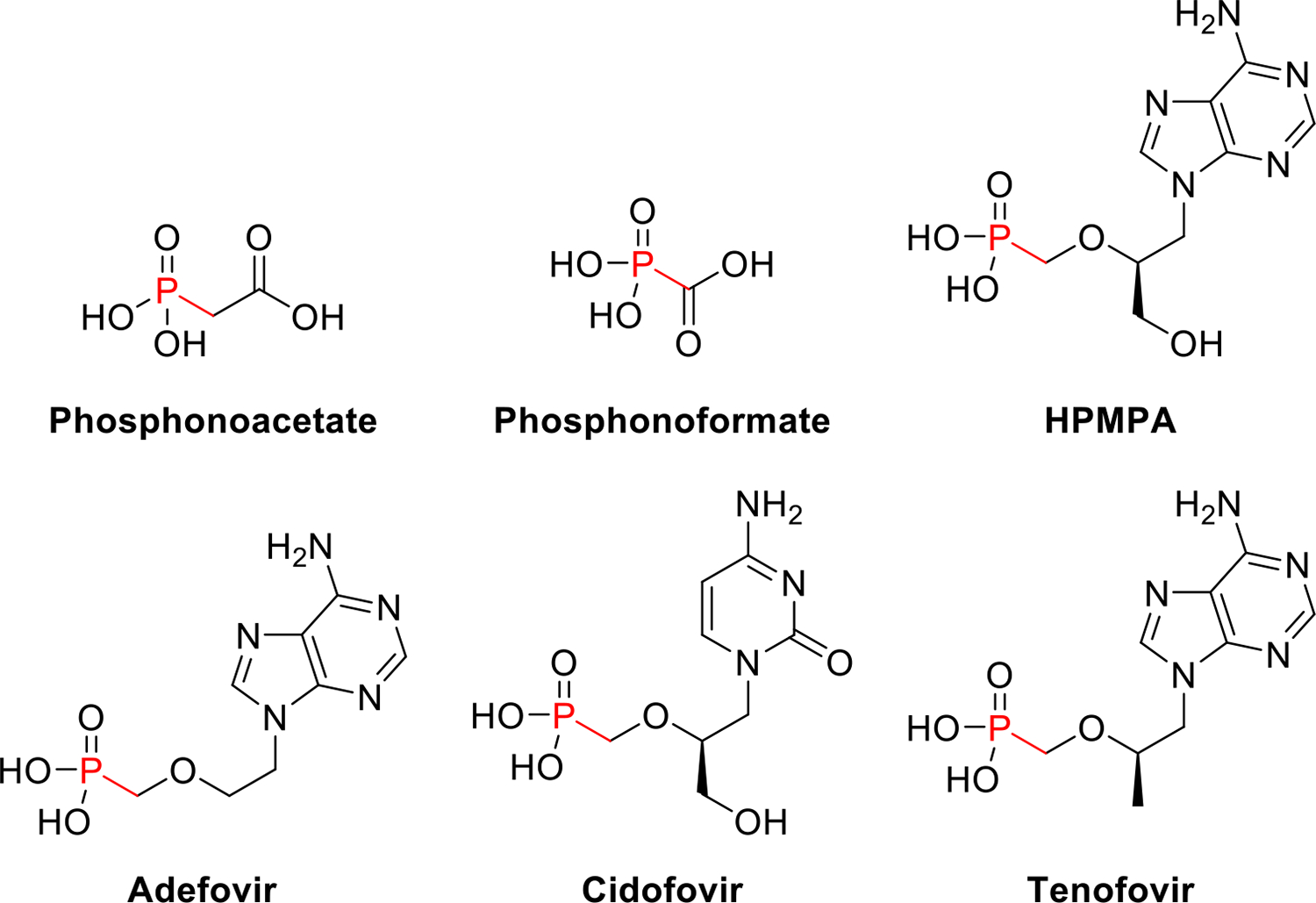
Structures of phosphonoacetate (PnA), phosphonoformate (PnF), the first acyclic nucleoside phosphonate (*S*)-9-(3-hydroxypropyl-2-methoxyphosphonyl)adenine (HPMPA), adefovir, cidofovir, and tenofovir.

Of the Pn drugs, the synthetic antiviral Pns have arguably had the greatest impact on human health. Development of the first Pn antivirals — also analogues of pyrophosphate — occurred in parallel with fosfomycin and the bisphosphonates. This began with the 1965 discovery of *p*-nitrobenzylphosphonate and dibenzylphosphonate as inhibitors of encephalomyocarditis virus replication [34]. Eight years later, during a “random testing of compounds,” researchers at Abbot Laboratories would later show phosphonoacetate (PnA, Figure 3) inhibited Herpes simplex virus (HSV) replication *in vitro* and *in vivo* [35]. Further investigation suggested that PnA interfered with DNA synthesis by binding to viral DNA polymerases at the pyrophosphate binding site [36].

Taking note of this isosterism, researchers at Michigan State University and Astra AB (now Astra-Zeneca) independently explored other pyrophosphate analogues, both finding that phosphonoformate (PnF, Figure 3) inhibits HSV [37,38]. Coincidentally, both PnA and PnF would be identified as NPs several decades later. Like PnA, the anti-HSV activity of PnF was attributed to the inhibition of viral DNA polymerases [39], but PnF was also found to inhibit many other polymerases [39]. Under the generic name foscarnet, PnF was officially approved by the FDA in 1991 and remains an important treatment for cytomegalovirus (CMV) and HSV in the immunocompromised [40].

These early antivirals led to the development of acyclic nucleoside phosphonates (ANPs), which arose from collaborations between Erik De Clercq and Antonín Holý. The three most prominent ANPs — cidofovir, adefovir, and tenofovir (Figure 3) — have enabled antiviral regimens for human immunodeficiency virus (HIV) and hepatitis B virus (HBV), among others [41]. The very first ANP, (*S*)-9-(3-hydroxypropyl-2-methoxyphosphonyl)adenine (HPMPA, Figure 3), has been referred to as a hybrid molecule between DHPA [(*S*)-9-(2,3-dihydroxypropyl)adenine] and PnF [41]. Compared to other antivirals, ANPs offer a broader spectrum of activity, a longer duration of action, and a lower risk of resistance — all of which have been attributed to the presence of the phosphonate moiety [41].

Much of the medicinal chemistry surrounding Pn prodrugs stems from studies with ANPs, as reviewed by Krečmerová et al. [42]. These compounds remain an active area of research against poxviruses and herpes-viruses lacking treatment, as well as protozoal infections such as trypanosomiasis [43]. Interestingly, cytidine monophosphate-5′-PnF (CMP-PnF) has been identified as an intermediate within phosphinothricin biosynthesis [44], and a similar nucleoside monophosphate-PnA (NMP-PnA) intermediate has been proposed within the biosynthesis of *O*-phosphonoacetic acid serine (*O*-PnAS) [6]. While the nucleotidylation of these intermediates is believed to serve as an activation step for further reactions, their bioactivity has yet to be defined. Nonetheless, it is tempting to speculate that Nature was indeed the first to synthesize antiviral nucleoside Pn due to the structural similarity of CMP-PnF and NMP-PnA to ANPs.

### Antibacterials

**Figure 4:**
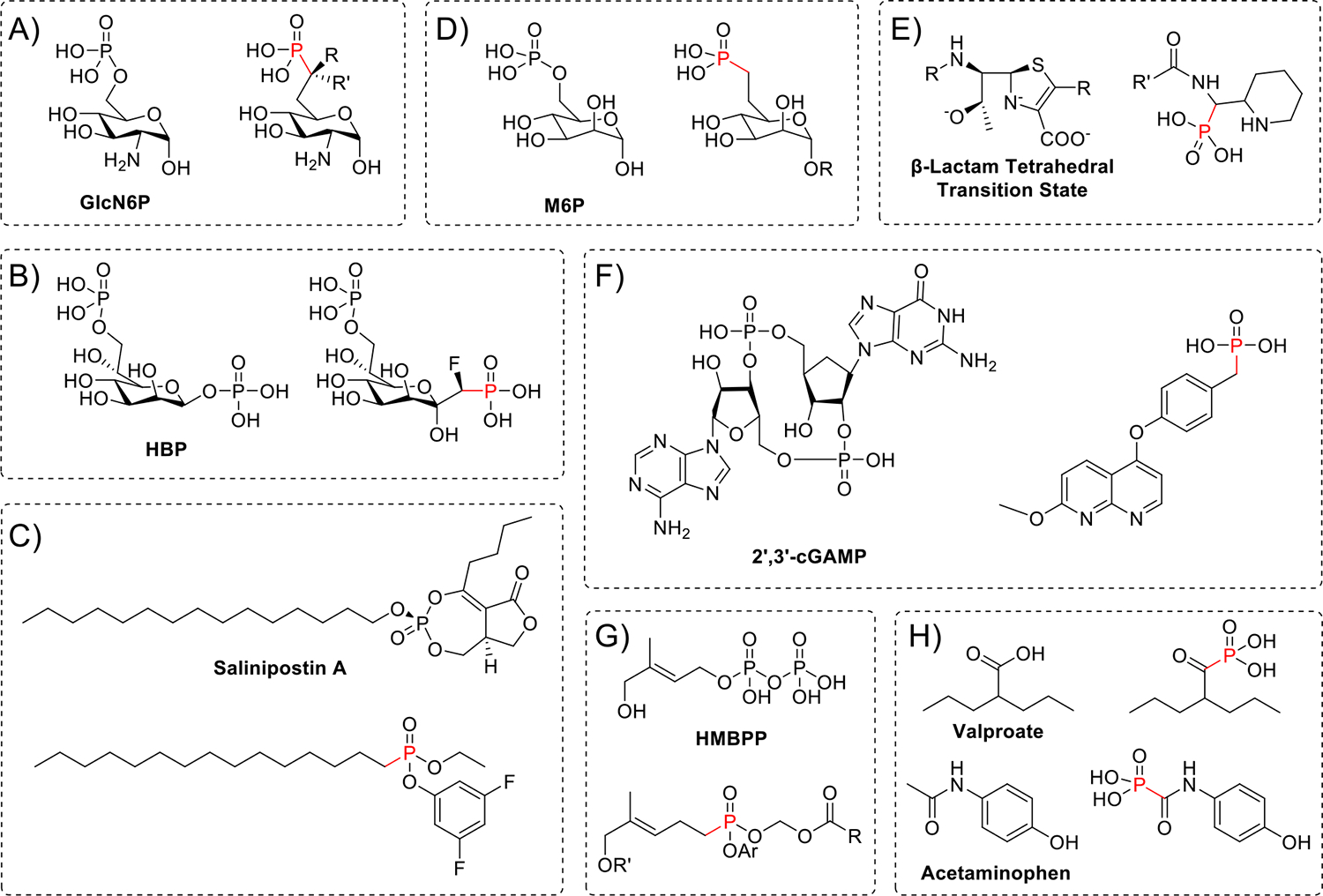
Synthetic inhibitors and their Pn analogues. **(a)** Pn analogues of glucosamine-6-phosphate (GlcN6P) were developed to activate the *glmS* riboswitch and inhibit cell wall biosynthesis (R and R′ = H, OH, or F). **(b)** Similarly, analogues of d-glycero-β-d-manno-heptose-1,7-bisphosphate (HBP) were developed to inhibit heptose biosynthesis. **(c)** A Pn analogue of salinipostin A was able to inhibit serine hydrolases via a distinct, novel mechanism. **(d)** Pn analogues of mannose-6-phosphate, the mannose-6-phosphonates (R = (CH_2_)_5_CONHNH_2_ or (CH_2_)_2_ONH_2_), targeted proteins to the lysosome for degradation. **(e)** Pn mimics of the tetrahedral transition state of metallo-β-lactamases to serve as inhibitors (R′ = various thiophenes [48]). **(f)** Pn inhibitors of ENPP1, which degrade 2′,3′-cyclic GMP-AMP (cGAMP), were able to enhance STING activity for cancer immunomodulation. **(g)** HMBPP is recognized by BTN3A1 to stimulate Vγ9Vδ2 T cell proliferation, and Pn analogues (Ar = Ph, *p*-IPr-Ph, 1-Nap; R′ = H, COCH_3_; R = Bn, CH_2_-Cy, oxane, CH_2_-oxane, *p*-MeO-Bn) were able to do the same. **(h)** Adding Pn moieties to acetaminophen and valproate improved the potency of acetaminophen while preserving the activity of valproate.

Recently, there has been a significant increase in the synthesis of Pn isosteres across divergent pathways for antimicrobial discovery. As activation of the bacterial *glmS* riboswitch by glucosamine-6-phosphate (GlcN6P) is known to interfere with cell wall biosynthesis, Silkenath et al. generated Pn analogues of GlcN6P (Figure 4a) [45]. Similarly, to target the heptose biosynthesis enzymes HldA and HldE, which are integral to lipopolysaccharide (LPS) biosynthesis, Pn analogues of d-glycero-β-d-manno-heptose-1,7-bisphosphate (HBP) were produced (Figure 4b) [46]. Starting with a known NP inhibitor of lipid-metabolizing serine hydrolases, mixed alkyl/aryl Pn bioisosteres of salinipostin A were synthesized, arriving at a new compound that killed *P. falciparum* through a mechanism distinct from that of the original NP (Figure 4c) [47]. And in a return to form, metallo-β-lactamase inhibitors were developed by designing a Pn which mimicked the tetrahedral transition state of hydrolyzed β-lactams (Figure 4e) [48], with the independent discovery of similar compounds through click-derived combinatorial chemistry [49]. While these new compounds are not technically NPs, the successful mimicry of primary metabolites is essential to their activity and offers a promising strategy for the development of new inhibitors.

### Immunomodulators

Outside of antimicrobials, Pn analogues have also recently been developed for immunomodulation. Pn inhibitors of ectonucleotide pyrophosphatase phosphodiesterase 1 (ENPP1) have been synthesized to enhance activation of stimulator of interferon gene (STING) [50]. The resulting cGAMP mimic significantly enhanced STING activity and synergized with radiotherapy in a murine model of pancreatic cancer (Figure 4f). To further explore the activation of Vγ9Vδ2 T cells for tumor immunotherapy, a Pn analogue of (*E*)-4-hydroxy-3-methylbut-2-enyl diphosphate (HMBPP) was developed (Figure 4g), which activated the butyrophilin receptor BTN3A1 to stimulate Vγ9Vδ2 T cell proliferation [51].

### Protein-targeted degradation agents

Pn isosteres have even been used to direct protein transport and degradation. Mannose-6-phosphate (M6P) modification of proteins is recognized by the M6P receptor (M6PR), which stimulates endocytosis into lysosomes. This transport signal was leveraged to treat Pompe disease in mice by synthesizing Pn analogues of M6P (Figure 4d) and grafting them to recombinant acid α-glucosidase [52]. Building on this concept, a mannose-6-phosphonate (M6Pn) glycopolypeptide that targeted the lysosome was designed and then fused with small molecules or antibodies to generate lysosome-targeting chimeras (LYTACs). In this manner, both secreted and membrane proteins could be targeted for degradation [53]. Recently, these ideas were further combined to directly functionalize monoclonal antibodies (mAbs) with M6Pn derivatives, allowing for more efficient degradation of soluble antigens such as tumor necrosis factor α (TNFα) [54].

### Other pharmaceutical isosteres

Interestingly, Pn derivatives can be effective even when their precise mechanisms of action are unknown. Pn analogues of paracetamol (acetaminophen, Tylenol) and valproate have been synthesized, replacing the methyl group of the former and hydroxyl of the latter (Figure 4h) [55]. The paracetamol derivative was four times more potent in preventing acetic acid-induced writhing in mice, while the Pn derivative of valproate was just as effective in the pentylenetetrazole-induced kindling mouse model. Careful dissection of the SAR in these cases may yield useful insights broadly applicable to medicinal chemistry as a whole.

## Conclusion and future perspectives

The molecular mimicry of Pn analogues has yielded both natural and synthetic therapeutics while advancing our overall understanding of metabolism. While the first Pn drugs to be clinically approved were synthetic compounds, the timeline for their development overlaps and intertwines with the development of fosfomycin, the first Pn NP drug. However, not everything “synthetic” is truly so. In 1979, Boezi noted that “phosphonoacetate is not a naturally occurring compound” [56] and the same was thought of phosphonoformate [39]. While both were chemically synthesized nearly one hundred years ago, their subsequent discovery as NPs suggests their production in Nature for millions of years before man [6,44].

Nearly every compound within this review is or has been inspired by an NP through mimicry of a primary or secondary metabolite. While chemical synthesis may initially appear as the simplest path to Pn therapeutics, the study of Pn NPs continues to yield even greater rewards. The bioactivity of NPs often reveals novel targets, as exemplified by fosmidomycin (the MEP pathway) and salinipostin A (serine hydrolases). Characterization of Pn biosynthesis has also yielded novel and synthetically useful enzymes. The mono-nuclear non-heme enzyme (*S*)-2-hydroxypropylphosphonic acid epoxidase (HPPE) has been the subject of intensive mechanistic investigations to elucidate the unusual manner in which HPP is converted into fosfomycin. This has empowered the recent engineering of HPPE into an enantioselective biocatalyst for *N*-fluoroamide directed C(*sp*^3^)-H fluorination [57].

NPs and their inhibitory activities have been selected for over tremendous evolutionary timescales. Despite the tremendous amount of effort devoted to fosmidomycin derivatives, “the *in vivo* efficacy has never been exceeded by an analog” [14]. And almost poetically, the search to develop a synthetic enolase inhibitor unwittingly converged on Nature’s existing solution, SF-2312 [22]. The discovery of additional Pn NPs should be prioritized as they continue to be a source of new chemical scaffolds, new drug targets, and novel enzymology while inspiring further synthetic analogues. And excitingly for the future, the vast majority of Pn NPs remain to be discovered [2].

## Data Availability

No data were used for the research described in the article.
